# Genetic mapping and genome-wide association study identify *BhYAB4* as the candidate gene regulating seed shape in wax gourd (*Benincasa hispida*)

**DOI:** 10.3389/fpls.2022.961864

**Published:** 2022-09-08

**Authors:** Chen Luo, Jinqiang Yan, Wenrui Liu, Yuanchao Xu, Piaoyun Sun, Min Wang, Dasen Xie, Biao Jiang

**Affiliations:** ^1^Vegetable Research Institute, Guangdong Academy of Agricultural Sciences, Guangzhou, China; ^2^Guangdong Key Laboratory for New Technology Research of Vegetables, Guangzhou, China; ^3^Institute of Vegetables and Flowers, Chinese Academy of Agricultural Sciences, Beijing, China

**Keywords:** wax gourd, seed shape, genetic mapping, genome-wide association study, YABBY, *BhYAB4*

## Abstract

Wax gourd is an important vegetable crop of the Cucurbitaceae family. According to the shape and structure of the seed coat, the seeds of the wax gourd can be divided into bilateral and unilateral. Bilateral seeds usually germinate quickly and have a high germination rate than unilateral seeds. Thereby, wax gourd varieties with bilateral seeds are more welcomed by seed companies and growers. However, the genetic basis and molecular mechanism regulating seed shape remain unclear in the wax gourd. In this study, the genetic analysis demonstrated that the seed shape of wax gourd was controlled by a single gene, with bilateral dominant to unilateral. Combined with genetic mapping and genome-wide association study, *Bhi04G000544* (*BhYAB4*), encoding a YABBY transcription factor, was identified as the candidate gene for seed shape determination in the wax gourd. A G/A single nucleotide polymorphism variation of *BhYAB4* was detected among different germplasm resources, with *BhYAB4^G^* specifically enriched in bilateral seeds and *BhYAB4^A^* in unilateral seeds. The G to A mutation caused intron retention and premature stop codon of *BhYAB4*. Expression analysis showed that both *BhYAB4^G^* and *BhYAB4^A^* were highly expressed in seeds, while the nuclear localization of BhYAB4^A^ protein was disturbed compared with that of BhYAB4^G^ protein. Finally, a derived cleaved amplified polymorphic sequence marker that could efficiently distinguish between bilateral and unilateral seeds was developed, thereby facilitating the molecular marker-assisted breeding of wax gourd cultivars.

## Introduction

Wax gourd [*Benincasa hispida* (Thunb.) Cogn., 2*n* = 2*x* = 24] is an important vegetable crop in the Cucurbitaceae family (Xie et al., 2019; Guo J. et al., 2020). Its fruits contain nutrients and metabolites that can treat various diseases and benefit the health of human beings (Gu et al., 2013). The storage period of the wax gourd is quite long, making it important in regulating the off-season sales of vegetables and ensuring the annual supply (Liu et al., 2018; Yan et al., 2021). According to the shape and structure of the seed coat, the seeds of the wax gourd can be divided into two types, unribbed seeds (also known as unilateral seeds) and ribbed seeds (also known as bilateral seeds). Seed shape is an important agronomic trait of wax gourd, which affects seed germination speed and germination rate. Unilateral seeds usually have different degrees of dormancy and germinate slowly and irregularly, while bilateral seeds germinate quickly and neatly.

In angiosperms, seeds are developed from the ovule and consist of the embryo, endosperm, and seed coat (Chaudhury et al., 2001). Fertilized egg cell and central cell differentiate into the embryo and the endosperm, respectively. The seed coat is originated from the maternal integuments, surrounding the developing embryo and endosperm (Chaudhury et al., 2001). In addition, the maternal seed coat is the outermost layer of the seed that can not only affect seed size and shape but also protect the seed (Radchuk and Borisjuk, 2014). Recent studies have revealed that ubiquitin-proteasome pathway, G-protein signaling, phytohormone signaling and homeostasis, mitogen-activated protein kinase signaling, and transcriptional regulators are involved in maternal control of seed development with a number of relevant genes being identified in *Arabidopsis* and rice (Li and Li, 2015; Li et al., 2019).

Seed size and shape of grain crops are important agronomic traits that affect yield and quality, which are important targets during domestication. Through map-based cloning and genome-wide association study (GWAS), many quantitative trait loci (QTLs) and genes for grain size and shape were isolated in rice, wheat, and maize (Li and Yang, 2017; Baye et al., 2022; Jiang et al., 2022). In cucurbits, seed size and shape are also important traits in breeding, which affect seed germination and the content of vegetable oil and proteins. Due to the rapid progress of genome sequencing and molecular biology in cucurbits, some seed size QTLs have been identified in major cucurbits, including watermelon, pumpkin, cucumber, and melon (Guo Y. et al., 2020).

The seed coat of *Arabidopsis* is composed of two layers of the outer integument and three layers of the inner integument (Haughn and Chaudhury, 2005). Ovule integument ontogeny has been extensively studied in *Arabidopsis*, and many genes involved in integument development have been identified (Gasser and Skinner, 2019). For example, *WUSCHEL* (*WUS*) and *AINTEGUMENTA* (*ANT*) promote integument initiation (Elliott et al., 1996; Klucher et al., 1996; Gross-Hardt et al., 2002). The YABBY transcription factor INNER NO OUTER (INO) and some KANADI family members are important regulators in specifying the adaxial–abaxial polarity of integuments (Leon-Kloosterziel et al., 1994; Villanueva et al., 1999; Eshed et al., 2001; McAbee et al., 2006).

*YABBY* genes encode a class of plant-specific transcription factors that are characterized by two conserved domains, the N-terminal C2C2 zinc finger domain and the C-terminal helix–loop–helix YABBY domain (Finet et al., 2016; Romanova et al., 2021). The *Arabidopsis YABBY* gene family contains six members, all of which specify the abaxial cell fate in lateral organs (Bowman and Smyth, 1999; Sawa et al., 1999; Siegfried et al., 1999; Villanueva et al., 1999; Sarojam et al., 2010). *INO* corresponds to *YABBY4* (*YAB4*), which is essential for the initiation and development of the ovule outer integument (Villanueva et al., 1999). The spatial expression pattern of *INO* is negatively regulated by several transcription factors, thus delimitating *INO* expression to the abaxial side of the outer integument (Balasubramanian and Schneitz, 2002; Meister et al., 2002). INO can directly interact with the co-repressors LEUNIG (LUG) and SEUSS (SEU) and the co-activator ADA2b/PROPORZ1 (PRZ1) to promote outer integument growth (Simon et al., 2017). In addition, *INO* is controlled by BRASSINAZOLE-RESISTANT 1 (BZR1) to modulate outer ovule integument development through the brassinosteroid pathway (Jia et al., 2020). A recent study revealed that INO also regulated iron loading into developing seeds by repressing the expression of *NATURAL RESISTANCE-ASSOCIATED MACROPHAGE PROTEIN 1* (*NRAMP1*), a gene encoding an iron transporter (Sun et al., 2021). In some plant species, *INO* genes are associated with the formation of seedless fruits. A spontaneous mutant of sugar apple, *Thai seedless* (*Ts*), lacks the ovule outer integument and produces seedless normal-sized fruits. Cloning analysis of the *INO* ortholog revealed that the *INO* locus is deleted in *Ts* plants compared with the wild-type and other members of this genus (Lora et al., 2011). The grape *INO* ortholog *VviINO* identified from the seeded cultivar successfully complements the ovule outer integument growth of *Arabidopsis ino* mutant and may be a candidate gene for the seedless trait (di Rienzo et al., 2021).

A previous study revealed that the heredity of wax gourd bilateral seed shape over unilateral seed shape was dominantly controlled by a single gene (Mi et al., 2021). In the present study, we combined genetic analysis, genetic mapping, and GWAS to identify the candidate gene for seed shape determination in the wax gourd. Our results will not only extend the functional study of seed shape determination gene in the wax gourd but also promote the marker-assisted breeding of wax gourd cultivars.

## Materials and methods

### Plant materials

**FIGURE 1:**
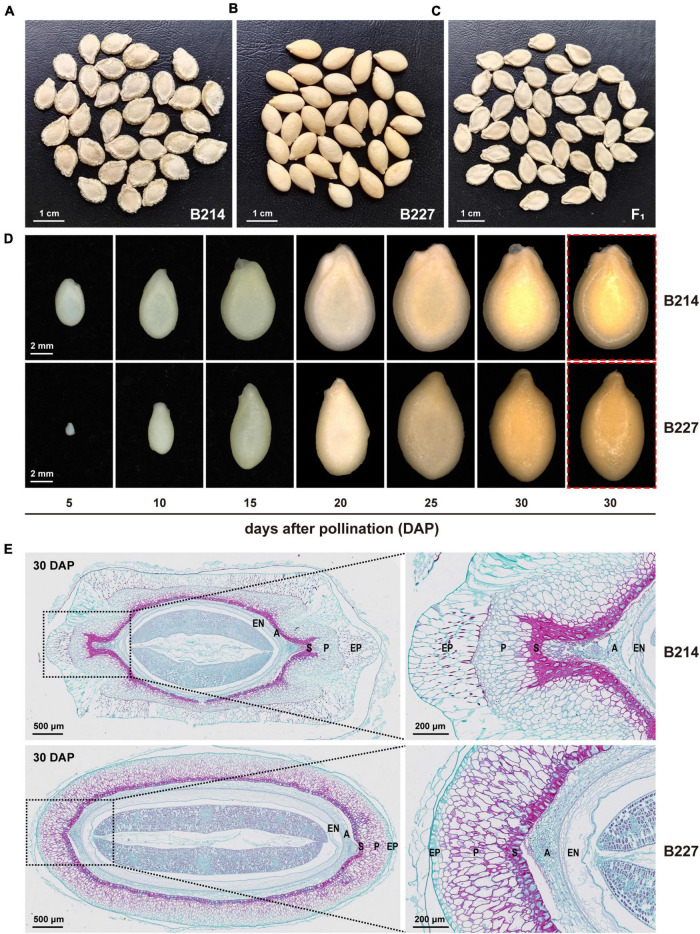
Phenotypic differences between wax gourd inbred lines B214 and B227. Mature seeds of **(A)** B214, **(B)** B227, and **(C)** F_1_ hybrid. **(D)** Seeds of B214 and B227 at different developmental stages. Red boxes indicate seeds with epidermal mucosa removed. **(E)** Transverse sections of the seeds of B214 and B227 at 30 days after pollination (DAP). EP, epidermis; P, parenchyma; S, sclerenchyma; A, aerenchyma; EN, endodermis.

Wax gourd inbred line B214 with bilateral seeds and B227 with unilateral seeds (Figures 1A,B) are derived from a Taiwan landrace and a cultivar ‘Sanshui Heipi Donggua,’ respectively. B214 (P_1_) was crossed with B227 (P_2_) to get F_1_ generation. The F_1_ was thereafter self-pollinated, backcrossed with P_1_ and P_2_ to obtain F_2_ generation, BC_1_P_1_, and BC_1_P_2_, respectively. All plant materials were grown according to standard agricultural practices with a 1.8 m line width and a 0.75 m row spacing in the research experimental field (23°23′N, 113°26′E; 20 m above sea level) of the Vegetable Research Institute, Guangdong Academy of Agricultural Sciences. The original seeds were provided by the Vegetable Research Institute, Guangdong Academy of Agricultural Sciences.

### Histological analysis

Seeds of the fruits 30 days after pollination (DAP) were taken out and immersed in formaldehyde-acetic acid-ethanol solution (37% formaldehyde:acetic acid:70% ethanol = 1:1:18). The samples were fixed over 24 h, then dehydrated with gradient alcohol and embedded in paraffin using xylene. Wax blocks were sliced into sections on the paraffin slicer. Subsequently, sections were rehydrated and stained using the safranin O/fast green staining method. Tissue sections were scanned using a Pannoramic MIDI digital slide scanner (3DHISTECH, Budapest, Hungary) and observed using the CaseViewer slide viewing application.^1^

### Preliminary genetic mapping

The F_2_ population and the high-density genetic map used in the present study were the same as previously described (Jiang et al., 2015). The F_2_ population included 140 individuals and the genetic map contained 12 linkage groups and 4,607 single nucleotide polymorphism (SNP) markers spanning 2,172.86 cM, with an average marker distance of 0.49 cM. The phenotypic data of seed shape in the F_2_ population was visually evaluated and recorded. Linkage analysis of the seed shape locus with specific-locus amplified fragment (SLAF) markers was performed with the Kosambi mapping function using the JoinMap 4.0 software.^2^ The threshold of significance (*P* = 0.05) for each marker after 1,000 permutations was set to 5.0.

### Fine mapping

A total of 2,112 F_2_ individuals were planted in 2021 for fine mapping in the experimental field of the Vegetable Research Institute, Guangdong Academy of Agricultural Sciences. Based on the initial mapping results, Marker78529 and Marker34358 were transferred to kompetitive allele-specific PCR (KASP) markers for recombinant selection from this large F_2_ population. The seed shape of all recombinants was observed during the mature fruit stage (around 50 DAP). To precisely narrow down the preliminary mapping interval, the resequencing data of two parental lines were analyzed to obtain SNPs that existed inside the interval. Based on the SNPs that existed, 16 pairs of primers evenly distributed were designed and used for PCR amplification of all the recombinants, parental lines, and F_1_ (detailed primer information see Supplementary Table 1). The amplification products were sent for sequencing to differentiate the B214 genotype, B227 genotype, and heterozygosity genotype. According to the genotype of recombinants derived by 16 pairs of primers, the recombination events were confirmed. Based on the recombination events and seed shape phenotype of the recombinants, the initial mapping region was further narrowed down.

### Genome-wide association study

Genome-wide association study was performed for seed shape using previously re-sequenced 146 wax gourd germplasm resources (Xie et al., 2019). A total of 2,237,614 SNPs with variation were used based on the previous analysis method (Xie et al., 2019), with missing rate ≤0.1 and minor allele frequency (MAF) ≥0.05, and the seed shape phenotypic data of 146 germplasm resources were used to perform the GWAS using the Emmax software (Kang et al., 2010). The Manhattan plot was drawn using the R software. GWAS threshold was set using *N* (the effective SNP number, *P* = 1/*N*). The effective SNP number was calculated using the Genetic type 1 Error Calculator (GEC) software (Li et al., 2012). Finally, the signals with *P* < 10^–6^ were considered as the significantly associated sites.

### Cloning and sequencing analysis of the candidate gene

The genomic sequence and cDNA sequence of the candidate gene were cloned using DNA from leaves and RNA from seeds, respectively. Primers were designed based on the Cucurbit Genomics Database^3^ using SnapGene software.^4^ PCR amplification was performed using the PrimeSTAR^®^ Max DNA Polymerase (Takara Bio, Shiga, Japan). Purified PCR products were ligated into *pEASY*^®^-Blunt Zero Cloning Vector (TransGen Biotech, Beijing, China) according to the manufacturer’s instructions. Then, ligation products were transformed into *Trans*1-T1 Phage Resistant Chemically Competent Cell (TransGen Biotech, Beijing, China). The positive clones identified by colony PCR were selected for sequencing analysis. Plasmids were extracted and sequenced at Sangon Biotechnology (Shanghai, China). The detailed primer information is listed in Supplementary Table 1.

### RNA extraction and quantitative real-time PCR

Total RNA was extracted from different wax gourd tissues and seeds at different developmental stages using a *TransZol* Up Plus RNA Kit (TransGen Biotech, Beijing, China). cDNAs were synthesized using a FastKing gDNA Dispelling RT SuperMix Kit (Tiangen Biotech, Beijing, China). Quantitative real-time PCR (qRT-PCR) was performed using the TB Green^®^ *Premix Ex Taq*™ II (Takara Bio, Shiga, Japan) on the CFX96™ Real-Time PCR Detection System (Bio-Rad, Hercules, CA, United States). Three biological replicates and three technical replicates were performed for each sample. The wax gourd ubiquitin gene (*Bhi10G000739*) was used as an internal reference to normalize the expression results, and the relative expression levels were calculated by the 2^–ΔΔCT^ method (Livak and Schmittgen, 2001). The detailed primer information is listed in Supplementary Table 1.

### Subcellular localization

The full-length coding sequences (CDSs) of *BhYAB4^G^* and *BhYAB4^A^* without the stop codon were fused with the green fluorescent protein (GFP) in the pSuper1300-GFP vector. The recombinant constructs were then transformed into *Agrobacterium tumefaciens* strain GV3101. GFP-fusion constructs and the nuclear localization marker (NLS-RFP) were co-infiltrated into *Nicotiana benthamiana* leaves. After 2 days infiltration, fluorescence signals were detected using a confocal laser scanning microscope (Zeiss LSM710, Jena, Germany). The detailed primer information is listed in Supplementary Table 1.

### Phylogenetic analysis

The YAB4 proteins of Cucurbitaceae crops were identified by BLASTP searches and sequences were downloaded from Cucurbit Genomics Database (see text footnote 3). Sequence of *Arabidopsis* YAB4 protein was downloaded from the Arabidopsis Information Resource.^5^ Sequence of tomato YAB4 protein was downloaded from the Phytozome Plant Genomics Resource.^6^ The obtained amino acid sequences were aligned with ClustalW. Then, a phylogenetic tree was constructed using the neighbor-joining method with 1,000 bootstrap replicates in the MEGA X software (Kumar et al., 2018). The accession numbers are listed in Supplementary Table 2.

### Development of derived cleaved amplified polymorphic sequence marker

One derived cleaved amplified polymorphic sequence (dCAPS) marker was developed based on the G/A SNP of *BhYAB4* gene in bilateral and unilateral seeds. Primers were designed using dCAPS Finder 2.0.^7^ PCR amplification was performed using genomic DNA of P_1_ (B214), P_2_ (B227), and 42 homozygous wax gourd germplasm resources. Then, PCR products were digested by *Sph*I restriction endonuclease and separated by native polyacrylamide gel. The detailed primer information is listed in Supplementary Table 1.

## Results

### Inheritance and phenotypic characterization of wax gourd seed shape

According to the shape and structure of the seed coat, the seeds of the wax gourd can be divided into bilateral (B214, P_1_) and unilateral (B227, P_2_) (Figures 1A,B). In this study, six-generation populations (P_1_, P_2_, F_1_, F_2_, BC_1_P_1_, and BC_1_P_2_) were obtained to investigate the inheritance of seed shape obtained by B214 and B227 (Table 1). The seed shape of all the F_1_ and BC_1_P_1_ individuals was bilateral (Figure 1C and Table 1). Among 160 BC_1_P_2_ individuals, 72 had bilateral seeds and 88 had unilateral seeds (Table 1), fitting with a 1:1 segregation ratio (χ^2^ = 0.802, *P* = 0.370). Among the 257 F_2_ individuals, 199 had bilateral seeds and 58 had unilateral seeds (Table 1). This segregation did not deviate from the 3:1 segregation ratio (χ^2^ = 0.419, *P* = 0.517). Taken together, these results indicated that the seed shape of wax gourd was controlled by a single gene, with bilateral dominant to unilateral.

**TABLE 1:** Segregation of seed shape in wax gourd segregation populations.

Population	No. of plants	Seed shape		Expected segregation ratio	Observed segregation ratio	χ^2^	*P*
		Bilateral	Unilateral				
P_1_ (B214)	40	40	0	1:0	1:0		
P_2_ (B227)	35	0	35	0:1	0:1		
F_1_	10	10	0	1:0	1:0		
BC_1_P_1_	139	139	0	1:0	1:0		
BC_1_P_2_	160	72	88	1:1	1:1.22	0.802	0.370
F_2_	257	199	58	3:1	3.43:1	0.419	0.517

To investigate the morphological differences between the bilateral seed and the unilateral seed, seeds of B214 and B227 at different developmental stages were observed under a stereo microscope (Zeiss SteREO Lumar V12, Jena, Germany) (Figure 1D). At 5 DAP, the ribs on the lateral side of the bilateral seeds appeared. As the seed developed, the ribs of the bilateral seeds became more prominent, while the surface of unilateral seeds remained smooth. To further characterize their differences, transverse sections of the 30 DAP seeds from B214 and B227 were analyzed. It could be seen that the seed coat was composed of five layers, including epidermis, parenchyma, sclerenchyma, aerenchyma, and endodermis (Figure 1E). There were significant differences in the shape and arrangement of the cells between bilateral and unilateral seeds (Figure 1E). Histological analysis showed that the ribs of bilateral seeds were caused by the outward projection of parenchyma and sclerenchyma on the lateral side, while the cells of unilateral seeds were arranged in ellipses, resulting in a smooth seed coat (Figure 1E). In addition, the epidermal cells of bilateral seeds are large and irregular, while those of unilateral seeds are small and regular (Figure 1E).

### Preliminary mapping of the candidate gene

**FIGURE 2:**
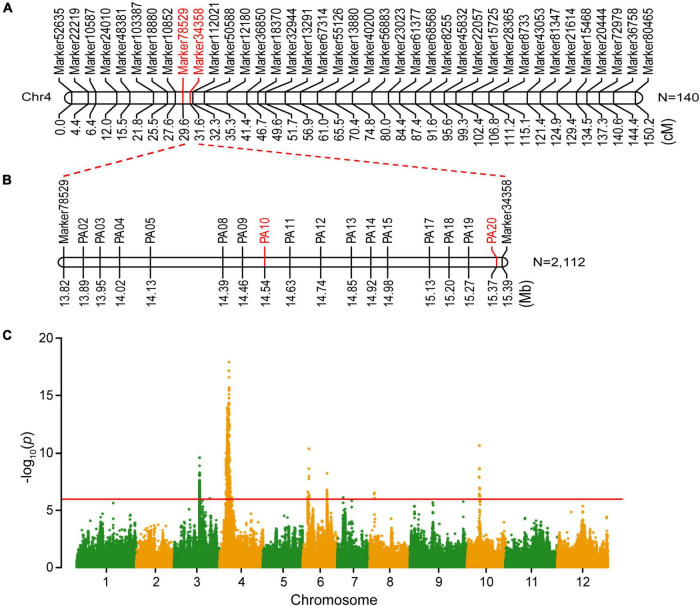
Genetic mapping and genome-wide association study (GWAS) of the seed shape gene in the wax gourd. **(A)** Initial genetic mapping of the seed shape locus. **(B)** Fine mapping of the seed shape locus. **(C)** Manhattan plots of seed shape through GWAS.

To genetically map the gene controlling seed shape of a wax gourd, 140 out of 257 F_2_ individuals were randomly selected for genetic map construction based on SLAF sequencing as previously described (Jiang et al., 2015). The genetic map was comprised of 12 linkage groups, with 4,607 SNP markers spanning 2,172.86 cM and an average distance of 0.49 cM between markers (Jiang et al., 2015). Based on the high-density genetic map and phenotypic character of seed shape, the seed shape locus of wax gourd was mapped to chromosome 4 (chr4), flanked by two markers, Marker78529 and Marker34358 (Figure 2A). The genetic distance between Marker78529 and Marker34358 was 2.0 cM. According to the physical position of these two flanking markers on the wax gourd reference genome, the seed shape locus is located in a 1.57-Mb region (from 13,821,462 to 15,392,673 bp), containing 52 genes.

### Fine mapping and genome-wide association study analysis of the candidate gene

To narrow the preliminary mapping region, a large F_2_ population including 2,112 individuals was used as plant materials. Marker78529 and Marker34358 were transferred to KASP makers to select recombinants from a large F_2_ population. In total, 18 recombinants were screened out and planted in the experimental field until harvest. Sixteen pairs of primers were developed to genotype the recombinants. Through genotype-phenotype joint analysis of the recombinants, the seed shape locus was finally mapped between PA10 and PA20 (from 14,548,295 to 15,378,554 bp), a 0.83-Mb region (Figure 2B). Based on the wax gourd reference genome, 18 annotated genes were located within this interval (Table 2). Further, according to the re-sequencing data, five SNP variations were identified in the intragenic region of four genes between B214 and B227, namely *Bhi04G000521*, *Bhi04G000522*, *Bhi04G000525*, and *Bhi04G000544*, and no variation was identified in other genes (Supplementary Table 3).

**TABLE 2:** Candidate annotated genes in the fine mapping region.

Gene ID	Physical position	Annotation
Bhi04G000521	14,579,905–14,581,988	Vignain-like
Bhi04G000522	14,628,375–14,629,085	Cysteine protease
Bhi04G000523	14,756,424–14,758,879	Polygalacturonase 1 beta-like protein 3
Bhi04G000524	14,767,748–14,772,053	Nucleic acid-binding, OB-fold-like protein
Bhi04G000525	14,801,757–14,806,932	Plant transposase
Bhi04G000526	14,819,491–14,819,788	AMSH-like ubiquitin thioesterase 2
Bhi04G000531	14,967,513–14,972,529	Type 2 DNA topoisomerase 6 subunit B-like isoform X4
Bhi04G000532	14,968,230–14,976,574	ADP-ribosylation factor
Bhi04G000533	14,993,867–15,008,867	Phosphatidylinositol 3-kinase
Bhi04G000534	15,089,262–15,095,093	Pectin lyase-like superfamily protein
Bhi04G000537	15,110,302–15,111,354	Pentatricopeptide repeat-containing protein
Bhi04G000538	15,111,628–15,112,299	Pentatricopeptide repeat-containing protein
Bhi04G000539	15,145,178–15,147,818	Cytochrome b-c1 complex, subunit 8 protein
Bhi04G000540	15,202,942–15,208,427	RNA-binding protein 48
Bhi04G000541	15,208,576–15,212,033	BOI-related E3 ubiquitin-protein ligase 1
Bhi04G000542	15,247,651–15,248,718	DNA-directed RNA polymerases I, II, and III subunit RPABC5
Bhi04G000544	15,260,460–15,262,407	Axial regulator YABBY 4-like
Bhi04G000545	15,295,640–15,299,032	Ubiquitin

In addition, GWAS analysis was performed for seed shape using previously resequenced 146 wax gourd germplasm resources (Xie et al., 2019). Finally, we identified a significant association signal at a threshold of −log_10_(*p*) > 15 on chromosome 4 (Figure 2C), overlapping with the gene mapping region. Analysis of the significant SNP revealed that the 15,261,579 bp site were correlated with the seed shape trait, which was located within the *Bhi04G000544* gene (Supplementary Table 4). Functional annotation revealed that *Bhi04G000544* encoded a member of the YABBY transcription factor family and was an ortholog of *Arabidopsis INO*/*YAB4*, a major regulator that promotes ovule outer integument development (Villanueva et al., 1999). Together, we predicted that *Bhi04G000544* was the candidate gene for seed shape determination in the wax gourd, and hereinafter designated as *BhYAB4*.

### Identification of *BhYAB4*

**FIGURE 3:**
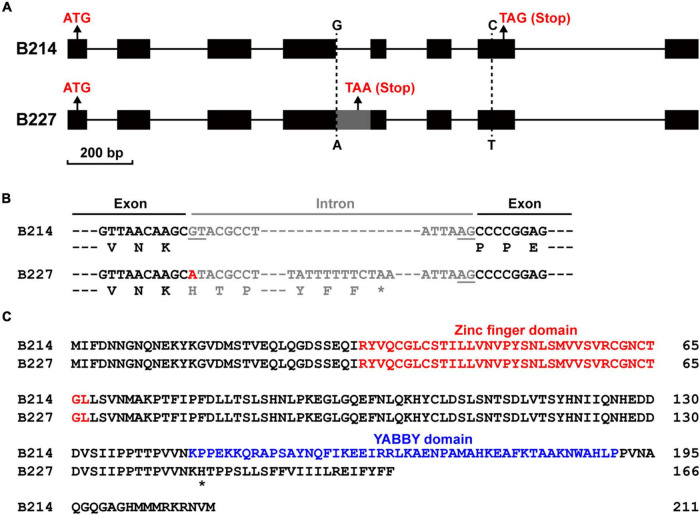
Mutation sites and amino acid changes in the *BhYAB4* gene. **(A)** Comparison of the gene structure of *BhYAB4* between two parental lines. Black boxes, exons; gray box, retained intron; solid lines, introns. **(B)** Comparison of the coding sequence and predicted protein sequence of *BhYAB4* between two parental lines. Exons and introns are shown as black and gray, respectively. Donor (GT) and acceptor (AG) intron splice sites are underlined. The G to A mutation is marked in red. Asterisk indicates the stop codon. **(C)** Alignment of the predicted protein sequence of *BhYAB4* in the parental lines. The zinc finger domain and the YABBY domain are presented in red and blue, respectively. Asterisk indicates the frameshift mutation site.

To confirm the sequence difference of *BhYAB4* between two parental lines, their genomic DNA and cDNA sequences were cloned. Sequence alignment results showed that the genomic sequence of *BhYAB4* contained two SNP variations between B214 and B227 (Figure 3A). A G/A SNP was found in the fourth intron, and this SNP was consistent with the GWAS results. Another C/T SNP was found in the seventh exon and resulted in the transformation of histidine (H) to tyrosine (Y) at the 202nd amino acid position. Surprisingly, a 107 bp insertion was found in the cDNA sequence of B227 compared with that of B214. The insertion in B227 was intron retention, and the G to A SNP mutation was in the first base of this intron (Figure 3A). Further, we analyzed the putative amino acid sequence and found that the retained intron in B227 caused frameshift mutation and premature stop codon (Figure 3B). Prediction of the protein structure revealed that BhYAB4^G^ protein in B214 contained the conserved N-terminal C2C2 zinc finger domain and C-terminal YABBY domain, while the frameshift mutation of BhYAB4^A^ protein in B227 led to the absence of the conserved YABBY domain (Figure 3C), which was crucial for the function of YABBY proteins.

### Expression analysis of *BhYAB4*

**FIGURE 4:**
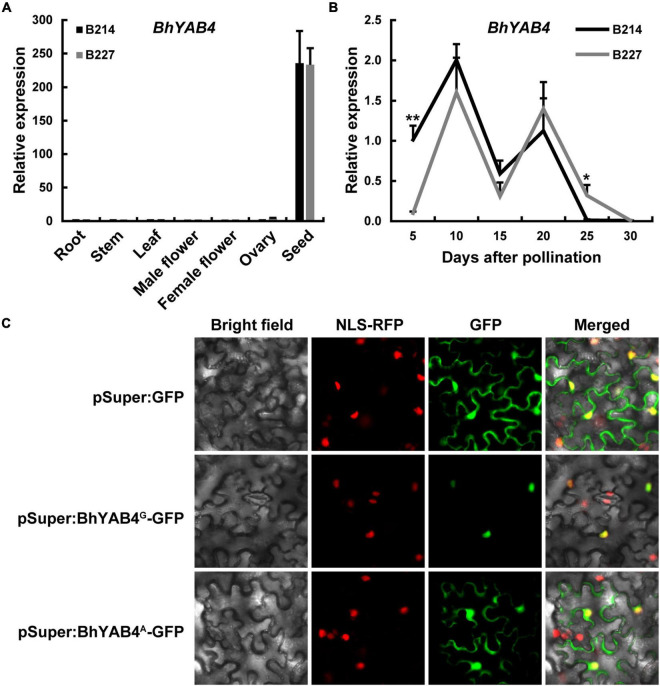
Expression pattern and subcellular localization of *BhYAB4*. **(A)** Relative expression of *BhYAB4* in different tissues of the wax gourd. Values are presented as means ± SD (*n* = 3). **(B)** Relative expression of *BhYAB4* in seeds at different developmental stages. Values are presented as means ± SD (*n* = 3). *P < 0.05, **P < 0.01 (Student’s *t*-test). **(C)** Subcellular localization of BhYAB4^G^ and BhYAB4^A^ proteins.

To further explore the expression pattern of the *BhYAB4* gene, qRT-PCR was performed. qRT-PCR results showed that *BhYAB4* was highly specifically expressed in the seeds of both B214 and B227 (Figure 4A). During different seed developmental stages, *BhYAB4* presented a similar expression trend between B214 and B227, and it was highly expressed in developing seeds at 10 DAP (Figure 4B), implying the important role of *BhYAB4* in regulating seed shape at this stage. However, the expression of *BhYAB4* was significantly higher in B214 than in B227 at 5 DAP, while was the opposite at 25 DAP (Figure 4B).

*BhYAB4* encodes a YABBY transcription factor, and the nuclear localization sequence (NLS) of YABBY proteins is located within the YABBY domain (Gross et al., 2018). To determine the subcellular localization of BhYAB4 protein, *BhYAB4^G^* and *BhYAB4^A^* genes were, respectively, fused with the GFP tag under the control of the Super promoter. Transient expression assays showed that BhYAB4^G^ protein was localized to the nucleus, while BhYAB4^A^ protein was localized to both the nucleus and plasma membrane (Figure 4C), indicating that BhYAB4 was a nuclear protein, and the absence of the YABBY domain disturbed its nuclear localization.

### Phylogenic analysis of BhYAB4 and its homologous proteins

**FIGURE 5:**
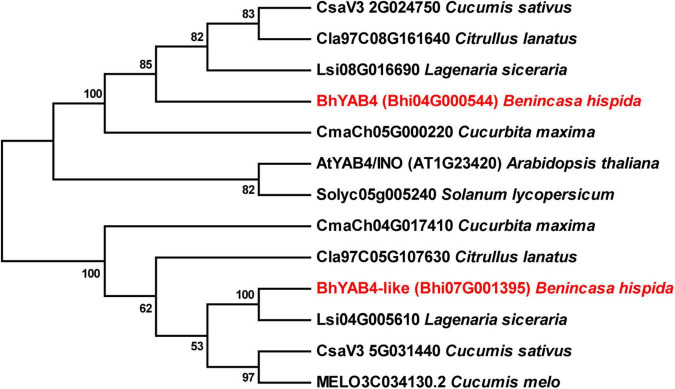
Phylogenetic analysis of BhYAB4 and its homologous proteins. The phylogenetic tree was constructed using YAB4 proteins from the wax gourd, *Arabidopsis*, tomato, and other cucurbits. Wax gourd YAB4 proteins are presented in red.

To dissect the relationship between BhYAB4 and other homologous proteins, we constructed a phylogenetic tree between wax gourd and other plant species, including cucumber, watermelon, melon, bottle gourd, pumpkin, *Arabidopsis*, and tomato. The generated phylogenetic tree revealed that BhYAB4 had a relatively close phylogenetic relationship with the YAB4 proteins of cucurbits (Figure 5). Interestingly, we found that two YAB4 proteins existed in the wax gourd, cucumber, watermelon, bottle gourd, and pumpkin (Figure 5). To further explore whether the two YAB4 proteins were functionally redundant in the wax gourd, we performed the qRT-PCR analysis of another *YAB4* gene, namely *BhYAB4*-like. Our results showed that *BhYAB4*-like was highly expressed in the ovary but showed low expression in the seed (Supplementary Figure 1), indicating that *BhYAB4*-like might have no function in regulating wax gourd seed shape.

### Development of derived cleaved amplified polymorphic sequence marker for seed shape determination

**FIGURE 6:**
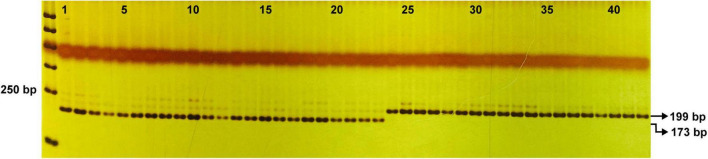
Validation of the efficiency of dCAPS marker in 42 wax gourd germplasm resources. 1–23, germplasm resources with unilateral seeds; 24–42, germplasm resources with bilateral seeds.

Based on the G/A SNP of the *BhYAB4* gene, one dCAPS marker was developed. After PCR amplification and *Sph*I enzyme digestion, the amplicon of B227 could be digested into 173 and 26 bp, but that of B214 could not be digested (Supplementary Figure 2). The F_2_ population was also validated using this marker, and the genotype was consistent with the phenotype. Further, 42 homozygous wax gourd germplasm resources were tested. Of the 42 germplasm resources, 23 had unilateral seeds with consistent brands as B227 and the other 19 had bilateral seeds with consistent brands as B214 (Figure 6 and Supplementary Table 5).

## Discussion

The seeds of cucurbits vary in shape, size, and color. Studies on seed-related traits of cucurbits mainly focus on seed size, and the selection of seed size depends on their intended uses (Guo Y. et al., 2020). For example, flesh-consumed watermelons often have small-seeded or seedless fruits, which can increase the edible part of the fruit flesh. Edible-seeded watermelons, which are used for seed consumption, often have larger seeds (Gong et al., 2022). In pumpkin, seeds are often processed into snacks and vegetable oil. Meru et al. (2018) showed that pumpkin seed size was positively correlated with oil content, but negatively correlated with seed protein content.

Bilateral seeds with fast germination speed and high germination rate as compared with unilateral seeds can benefit the production of the wax gourd. Therefore, seed shape is an important agronomic trait in the wax gourd, which is a target of selection during wax gourd breeding. However, few studies have reported the seed shape difference in cucurbits, including wax gourd.

### *BhYAB4* is a candidate gene for seed shape determination in wax gourd

Previously, a study analyzed the inheritance of fruit and seed traits of the wax gourd. The results showed that the seed shape with bilateral or unilateral was controlled by a single gene (Mi et al., 2021). In the present study, we confirmed that the seed shape of wax gourd was controlled by a single gene, with bilateral dominant to unilateral (Figures 1A–C and Table 1). Genetic mapping identified four candidate genes with SNP variations (Supplementary Table 3), and expression analysis showed that only *BhYAB4* was highly expressed in seeds (Figure 4A and Supplementary Figure 3). Combined with GWAS analysis, *BhYAB4* was determined as the seed shape determination gene (Figure 2C), and a G/A SNP was found among different germplasm resources, with *BhYAB4^G^* specifically enriched in bilateral seeds and *BhYAB4^A^* in unilateral seeds (Figure 3A and Supplementary Table 4). Because the wax gourd reference genome was based on B227, the G to A SNP was defined as the exon region of *BhYAB4* in B227 previously. We found that this SNP variation was in the first base of the fourth intron in B214 according to the original *BhYAB4* gene encoding the complete YABBY protein sequence (Figure 3). Previous studies have shown that intronic GT-AG dinucleotides are highly conserved at the intron splice sites, and its mutation will compromise pre-mRNA splicing (Pucker and Brockington, 2018; Xue et al., 2018). In addition, the alignment of the *YAB4* sequences of wax gourd and other cucurbits showed that the same site is G base in other cucurbits (Supplementary Figure 4). The G to A mutation of *BhYAB4* affected the intron splice site and retained the intron, finally resulting in a 107 bp insertion in the cDNA sequence of B227 compared with that of B214 (Figure 3A).

Because of the intron retention, a premature stop codon was induced, leading to a truncated BhYAB4^A^ protein lacking the conserved YABBY domain. The YABBY family protein CRABS CLAW (CRC) was localized to the nucleus, and the nuclear localization of CRC requires the C-terminal YABBY domain (Gross et al., 2018). Consistently, our results showed that the presence of the YABBY domain of the BhYAB4 protein was essential for its nuclear localization (Figure 4C). In addition, the YABBY domain is necessary for DNA binding and protein interaction (Simon et al., 2017; Gross et al., 2018), thus the truncated BhYAB4^A^ protein of wax gourd may occupy incomplete function, indicating the crucial role of full-length BhYAB4^G^ protein in regulating wax gourd seed shape.

Based on the G/A SNP of the *BhYAB4* gene, a dCAPS marker was developed for seed shape determination. The dCAPS marker is co-segregated with seed shape phenotype (Figure 6 and Supplementary Table 5), which can be used for marker-assisted selection in the future. Interestingly, we also found that most wild and landrace wax gourds have bilateral seeds, while cultivated wax gourds have both bilateral and unilateral seeds (Supplementary Table 4), implying that mutation of the *BhYAB4* gene resulted in the change of seed shape from bilateral to unilateral during wax gourd improvement process.

### Divergent roles of YAB4 homologs in different species

YABBY transcription factors are well known for their roles in specifying abaxial cell fate in lateral organs (Finet et al., 2016; Zhang et al., 2020; Romanova et al., 2021). In *Arabidopsis*, YAB4/INO plays a central role during ovule outer integument development (Villanueva et al., 1999). In sugar apple and grape, the *INO* genes are associated with the formation of seedless fruits (Lora et al., 2011; di Rienzo et al., 2021). In addition, the tomato *SlINO* gene is associated with fruit shape (Han et al., 2015). A study identified two *INO* homologs in most cucurbits, which may be caused by gene duplication during evolution (Yin et al., 2022). However, perhaps due to the lack of high-quality reference genomes, only one or no *INO* homolog has been identified in some cucurbits. In cucumber, the two *INO* homologs were differently expressed in different tissues (Yin et al., 2022), and our results showed that the two wax gourd *YAB4* homologs also exhibited different expression patterns (Figure 4A and Supplementary Figure 1), suggesting that the two *YAB4* homologs in cucurbits may have different biological functions.

Here, we identified *BhYAB4* as the candidate gene for wax gourd seed shape determination (Figure 2), while little is known about its regulatory mechanism. Recently, an efficient genetic transformation system was established for cucurbits (Xin et al., 2022), which will accelerate gene function validation in the related plant species. In future studies, combing with the optimized genetic transformation method, the biological function of *BhYAB4* will be elucidated through genome editing and overexpression technologies. Meanwhile, the molecular mechanism of *BhYAB4* in regulating seed shape also needs to be explored.

## Data availability statement

The datasets presented in this study can be found in online repositories. The names of the repository/repositories and accession number(s) can be found in the article/Supplementary material.

## Author contributions

BJ, CL, and JY: conceptualization and writing—review and editing. CL, JY, WL, YX, PS, and MW: data collection. CL, JY, WL, and YX: formal analysis and investigation. CL and JY: writing—original draft preparation. DX and BJ: resources. BJ: supervision. All authors read and approved the final manuscript.

## Footnotes

1
https://www.3dhistech.com/solutions/caseviewer/

2
https://www.kyazma.nl/index.php/JoinMap/

3
http://cucurbitgenomics.org/

4
https://www.snapgene.com/

5
https://www.arabidopsis.org

6
https://phytozome-next.jgi.doe.gov

7
http://helix.wustl.edu/dcaps/dcaps.html
