# Supplementary material for: Genetic mapping and genome-wide association study identify BhYAB4 as the candidate gene regulating seed shape in wax gourd (Benincasa hispida)
Source: Front Plant Sci. 2022 Sep 8;13:961864. doi: 10.3389/fpls.2022.961864 (PMC9493316; doi:10.3389/fpls.2022.961864)
Supplement: Supplementary file 1 [file Data_Sheet_1.PDF]

Supplementary Material

1 Supplementary Figures and Tables

1.1 Supplementary Figures

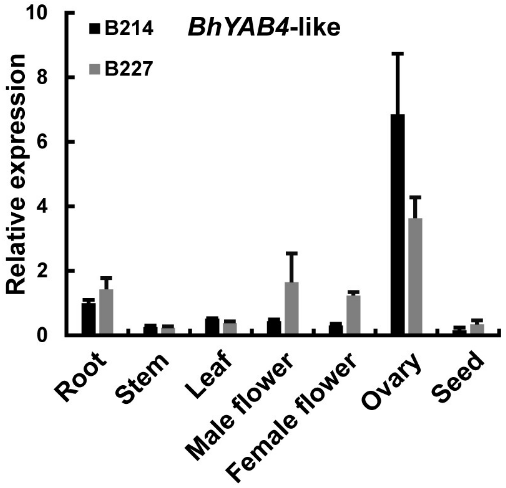

Supplementary Figure 1. Expression pattern of *BhYAB4*-like.

Relative expression of *BhYAB4*-like in different tissues of wax gourd. Values are presented as means ±SD (*n*=3).

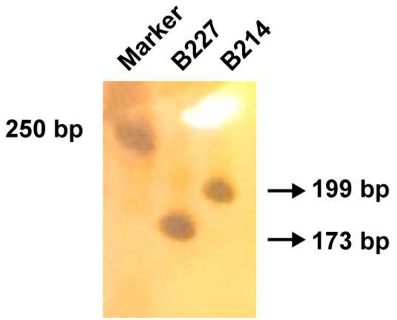

Supplementary Figure 2. Native polyacrylamide gel image shows the bands of B214 and B227 using the dCAPS marker.

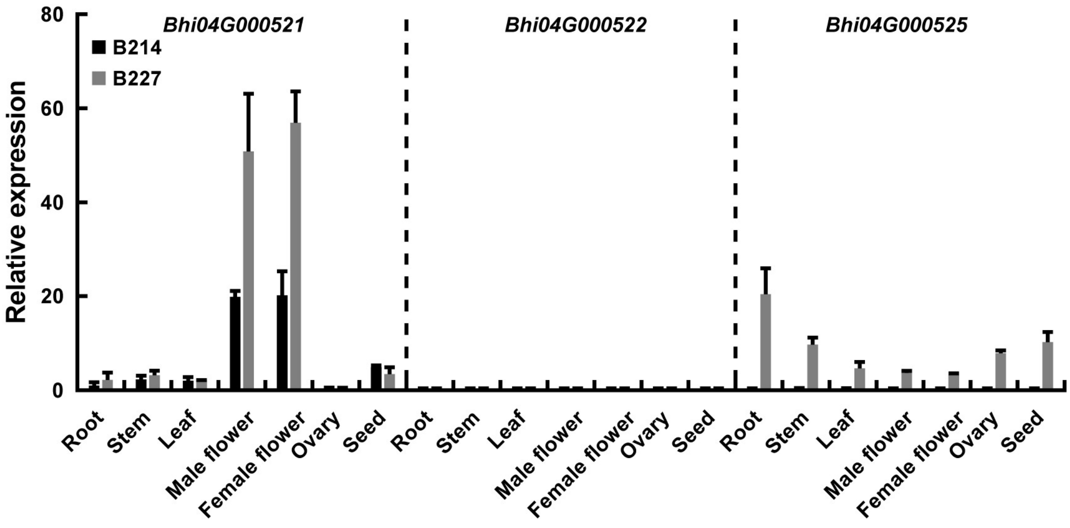

Supplementary Figure 3. Expression pattern of *Bhi04G000521*, *Bhi04G000522*, and *Bhi04G000525*.

Relative expression of *Bhi04G000521*, *Bhi04G000522*, and *Bhi04G000525* in different tissues of wax gourd. Values are presented as means ±SD (*n*=3).

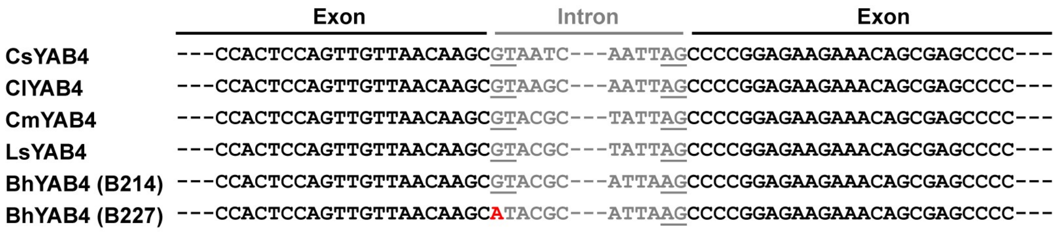

Supplementary Figure 4. Comparison of the genomic sequence of *YAB4* in wax gourd and other cucurbits.

Exons and introns are shown in black and gray, respectively. Donor (GT) and acceptor (AG) intron splice sites are underlined. The G to A mutation in wax gourd line B227 is in red. Cs, *Cucumis sativus.* Cl, *Citrullus lanatus*. Cm, *Cucurbita maxima*. Ls, *Lagenaria siceraria*. Bh, *Benincasa hispida*.

1.2 Supplementary Tables

Supplementary Table 1. Primer information in this study.

Primer name	Purpose	Forward sequence	Reverse sequence
Marker78529	Genotyping	A1:GAAGGTCGGAGTCAACGGATTTTGGGATATCCAACATCACATAGA<br>A2:GAAGGTGACCAAGTTCATGCTTGGGATATCCAACATCACATAGG	ACTTCTGTGATTCTGTATGGATTGAA
Marker34358	Genotyping	A1:GAAGGTCGGAGTCAACGGATTTTGAAGTGCATACTATTTATGTAAGTTCTA<br>A2:GAAGGTGACCAAGTTCATGCTTGAAGTGCATACTATTTATGTAAGTTCTT	TTAATAAGTTTTCTTATTTCCTTATTTTAGC
PA02	Genotyping	ATATAACACCGTTCATAATAGAGACTTGCATC	CTTAAGGGAGTAATTTATCTACTTACCCCGAC
PA03	Genotyping	CTTGTTTGTGTTTGAAGGATTGTTGTAATGTA	GCATTTGTCACATTTATTGTTACCTTTACCAT
PA04	Genotyping	TGCAAGTTTGATTTAATTCTTTTGCATGTTTG	CGTTAAACGATACCCAAGCATCTGATA
PA05	Genotyping	TAATTCCTAGAGACAATTGGAAGTTGAAGATG	GAAAGTTTATATGCATTTCATAAGGTGTGCTT
PA08	Genotyping	TCTCTCATATTATCTTTCCCTCCCCATAATTC	GAATCTGCTCAACCCATCACCTTAACTC
PA09	Genotyping	GATCTTTCCAAAGACATAGTGACAGAATTACC	CAGAAAATTTTGGGTTGGGTTGGGTTG
PA10	Genotyping	CTCCAAAGAATAAGAAATCGTCCATAGATACT	CTTCATTGATTAAATGCTTGATAGATTGGCTG
PA11	Genotyping	CAATGTGAGTGGTTTCTCCTTTTACTCTTTTA	CATCCCATTCAAGGTTTTTCCTTCTCAAA
PA12	Genotyping	GGTTTAGTACTTTATTCGGTCCCTATATTTCA	GAAAAGTTGGATTTCAAGGAGTGTCTTTTTAG
PA13	Genotyping	GATGAAGTAAAGGATCATCGGGAGTTACTTG	CACCGGTTTCATGTGTTGAGAGTTC
PA14	Genotyping	TCAAACTTCTCCTCATACTCCTATACTGTCAT	CCGCTTTTGTTCTCATCGATAATGCTAAG
PA15	Genotyping	GATGCATCAGTTTTCGTCTTCATTTCG	TGAATTTACATCTTTACAAATCTTGAAATGGC
PA17	Genotyping	ATTATCCTTAGATTTGCATAGGTGAAGATTGG	GATCATCAAATCGAGTTCTTCTCAAGCTAATG
PA18	Genotyping	AAACTGTTTGATGTTTTATTTCGAAGGTGAAT	GGAGGAATAAAGTCCAGTGATTTGGTTTAATA
PA19	Genotyping	ACAAAGATGGGATTAAAGACATGAATCAAGAA	GGCTATGAACAAGAGAATCATGACAAACTTTA

PA20	Genotyping	GACCCAAACGATAGTTTAAGTGAACGAATCA	ATTCACAATGCATTACTGATATGGACCAGTTT
BhYAB4-clone	Gene cloning	AAGGAAACCATTAATTGAAAGGGAA	TTGGTGTGTTCGTGTCAGGATG
BhYAB4-CDS-clone	Gene cloning	ATGATATTTGATAATAATGGCAACCAAA	CTACATTACGTTTCTCTTCCTCATC
BhYAB4-qRT	qRT-PCR	TCTGAACAGATTCGCTATG	AGAGGGAAGTGAGAAGATC
BhYAB4-like-qRT	qRT-PCR	AAAGACAGCGTGCTCCATCA	GCTGCTGTTCTAAAGGCTTCC
Bhi04G000521-qRT	qRT-PCR	TAATGCAAGCCGTCGCAAAC	GCTCTGTTCCGCAGTATCCA
Bhi04G000522-qRT	qRT-PCR	GAAGCTGTTGGTGGTCGGTG	CGTCTTCCTCCATTCCTAGCC
Bhi04G000525-qRT	qRT-PCR	TGACAACTTCAACAACTAATGTGG	TGTACGAGTCCCACCAAACC
BhUBQ-qRT	qRT-PCR	CCTAACTGGGAAGACGAT	CAAGACCAAGTGAAGGGT
BhYAB4^G-GFP	Subcellular localization	ATCGACTCTAGAAAGCTTATGATATTTGATAATAATGGCAACCA	GGTACCGGATCCACTAGTCATTACGTTTCTCTTCCTCATC
BhYAB4^A-GFP	Subcellular localization	ATCGACTCTAGAAAGCTTATGATATTTGATAATAATGGCAACCA	GGTACCGGATCCACTAGTGAAAAAATAAAAAATTTCACGTAGAA
BhYAB4-dCAPS	dCAPS	CAATGTGTCAGCCAAAGGAG	AATGATAAGAGAGATGGAGGCGCA

Supplementary Table 2. Accession numbers of genes in this study.

Species	Gene name	Accession number
Arabidopsis thaliana	*AtYAB4/INO*	AT1G23420
Benincasa hispida	*BhUBQ*	Bhi10G000739
Benincasa hispida	*BhYAB4*	Bhi04G000544
Benincasa hispida	*BhYAB4*-like	Bhi07G001395
Cucumis sativus	*CsYAB4*	CsaV3_2G024750
Cucumis sativus	*CsYAB4*-like	CsaV3_5G031440
Citrullus lanatus	*ClYAB4*	Cla97C08G161640
Citrullus lanatus	*ClYAB4*-like	Cla97C05G107630
Cucurbita maxima	*CmYAB4*	CmaCh05G000220
Cucurbita maxima	*CmYAB4*-like	CmaCh04G017410
Lagenaria siceraria	*LsYAB4*	Lsi08G016690
Lagenaria siceraria	*LsYAB4*-like	Lsi04G005610
Cucumis melo	*CmeYAB4*	MELO3C034130.2
Solanum lycopersicum	*SlINO*	Solyc05g005240

Supplementary Table 3. SNP variations in the intragenic region of the candidate genes between B214 and B227.

**Chr**	**Position**	**B227 (Reference)**	**B214**	**Exon or intron**	**Gene ID**
chr4	14,580,892	A	G	intron	Bhi04G000521
chr4	14,628,997	G	A	exon	Bhi04G000522
chr4	14,802,155	A	G	exon	Bhi04G000525
chr4	15,261,096	A	G	exon	Bhi04G000544
chr4	15,261,579	T	C	exon (intron retention)	Bhi04G000544

Supplementary Table 4. Seed shape and SNP variations of the 146 re-sequenced wax gourd germplasm resources.

Sample name	Cultivar	Origin	Group[1]	Seed shape	*BhYAB4* G/A[2]
Bhi-1	BF13	Punjab, India	W	Bilateral	G
Bhi-2	BF19	Punjab, India	W	Bilateral	G
Bhi-3	BF1	Rajasthan, India	W	Bilateral	G
Bhi-4	B260-c	Fukushima-ken, Japan	W	Bilateral	G
Bhi-5	B260-a	Fukushima-ken, Japan	W	Bilateral	G
Bhi-6	HB260-a	Self created material	W	Bilateral	G
Bhi-7	HB260-b	Self created material	W	Bilateral	G
Bhi-8	B260-b	Fukushima-ken, Japan	W	Bilateral	G
Bhi-9	HF3-c	Self created material	W	Bilateral	G
Bhi-10	HF3-b	Self created material	W	Bilateral	R
Bhi-11	HF3-a	Self created material	W	Bilateral	R
Bhi-12	F3	Self created material	W	Unilateral	A
Bhi-13	HF3-d	Self created material	W	Bilateral	R
Bhi-14	BN35	Jinghong, Yunnan, China	L	Bilateral	G
Bhi-15	BN32	Jinghong, Yunnan, China	L	Bilateral	G
Bhi-16	BN1603	Puer, Yunnan, China	L	Bilateral	G
Bhi-17	BN1612	Puer, Yunnan, China	L	Bilateral	G
Bhi-18	BN47	Jinghong, Yunnan, China	L	Bilateral	G
Bhi-19	BN34	Jinghong, Yunnan, China	L	Bilateral	G
Bhi-20	BN1615	Lancang, Yunnan, China	L	Bilateral	G
Bhi-21	BN55	Jinghong, Yunnan, China	L	Bilateral	G
Bhi-22	BN40	Jinghong, Yunnan, China	L	Bilateral	G
Bhi-23	BN1610	Puer, Yunnan, China	L	Bilateral	G
Bhi-24	B214	Taiwan, China	L	Bilateral	G
Bhi-25	S15	Taiwan, China	L	Bilateral	G
Bhi-26	BN1620	Jinghong, Yunnan, China	L	Bilateral	G
Bhi-27	B421	Quang Tri, Tinh, Vietnam	L	Bilateral	G
Bhi-28	P90	Nanning, Guangxi, China	C1	Bilateral	G
Bhi-29	P73	Haikou, Hainan, China	C1	Bilateral	G
Bhi-30	P89	Suixi, Guangdong, China	C1	Bilateral	G
Bhi-31	BN10	Kunming, Yunnan, China	L	Bilateral	G
Bhi-32	P84-1	Xiangyang, Hubei, China	C1	Bilateral	G
Bhi-33	P126	Huangmei, Hubei, China	C1	Bilateral	G
Bhi-34	B242	Beijing, China	C1	Bilateral	A
Bhi-35	P74	Hefei, Anhui, China	C1	Bilateral	G
Bhi-36	P72	Suqian, Jiangxi, China	C1	Bilateral	G
Bhi-37	P129	Nanchang, Jiangxi, China	C1	Bilateral	G
Bhi-38	B318	Nanjing, Jiangsu, China	C1	Unilateral	A
Bhi-39	P91	Haikou, Hainan, China	C1	Bilateral	G
Bhi-40	B397	Nanjing, Jiangsu, China	C1	Bilateral	G
Bhi-41	BN48	Baoshan, Yunnan, China	L	Unilateral	A
Bhi-42	GL-4	Guangzhou, Guangdong, China	C1	Bilateral	G
Bhi-43	C2-6-2	Self created material	C1	Bilateral	G

Bhi-44	A39FA	Self created material	C1	Bilateral	G
Bhi-45	A39	Guangzhou, Guangdong, China	C1	Bilateral	G
Bhi-46	B266	Tianjing, China	C1	Bilateral	G
Bhi-47	C4-3-1A	Self created material	C1	Bilateral	G
Bhi-48	C30	Self created material	C1	Bilateral	G
Bhi-49	LY1	Ningyang, Shandong, China	C1	Bilateral	G
Bhi-50	P96	Dongguan, Guangdong, China	C1	Unilateral	R
Bhi-51	P33	Taishan, Guangdong, China	C1	Unilateral	A
Bhi-52	P97	Taishan, Guangdong, China	C1	Unilateral	A
Bhi-53	B507	Changsha, Hunan, China	C1	Bilateral	G
Bhi-54	H10	Jiangmen, Guangdong, China	C1	Bilateral	G
Bhi-55	H9	Jiangmen, Guangdong, China	C1	Bilateral	G
Bhi-56	H1	Foshan, Guangdong, China	C1	Bilateral	G
Bhi-57	P75	Suixi, Guangdong, China	C1	Bilateral	G
Bhi-58	B338	Conghua, Guangdong, China	C1	Unilateral	-
Bhi-59	P86	Dongguan, Guangdong, China	C1	Unilateral	R
Bhi-60	BS529	Sanshui, Guangdong, China	C2	Bilateral	G
Bhi-61	B483	Changsha, Hunan, China	C2	Bilateral	R
Bhi-62	B372	Chengdu, Sichuan, China	C2	Bilateral	A
Bhi-63	B442	Changsha, Hunan, China	C2	Bilateral	R
Bhi-64	BS469	Taishan, Guangdong, China	C2	Bilateral	G
Bhi-65	B258	Shantou, Guangdong, China	C2	Unilateral	A
Bhi-66	B501	Jiexi, Guangdong, China	C2	Unilateral	A
Bhi-67	B503	Jiexi, Guangdong, China	C2	Unilateral	A
Bhi-68	B496	Jiexi, Guangdong, China	C2	Unilateral	A
Bhi-69	B498	Jiexi, Guangdong, China	C2	Unilateral	A
Bhi-70	B486	Jiexi, Guangdong, China	C2	Unilateral	A
Bhi-71	B450	Dongguan, Guangdong, China	C2	Unilateral	A
Bhi-72	B489	Jiexi, Guangdong, China	C2	Unilateral	A
Bhi-73	B488	Jiexi, Guangdong, China	C2	Unilateral	A
Bhi-74	B494	Jiexi, Guangdong, China	C2	Unilateral	A
Bhi-75	B491	Jiexi, Guangdong, China	C2	Unilateral	A
Bhi-76	B490	Jiexi, Guangdong, China	C2	Unilateral	A
Bhi-77	B487	Jiexi, Guangdong, China	C2	Unilateral	A
Bhi-78	B504	Jiexi, Guangdong, China	C2	Unilateral	A
Bhi-79	B500	Jiexi, Guangdong, China	C2	Unilateral	A
Bhi-80	B517	Jiexi, Guangdong, China	C2	Unilateral	A
Bhi-81	B502	Jiexi, Guangdong, China	C2	Unilateral	A
Bhi-82	B495	Jiexi, Guangdong, China	C2	Unilateral	A
Bhi-83	B499	Jiexi, Guangdong, China	C2	Unilateral	A
Bhi-84	B264	Guangzhou, Guangdong, China	C2	Unilateral	A
Bhi-85	B280-2	Dafeng, Jiangsu, China	C2	Unilateral	A
Bhi-86	B519	Yingde, Guangdong, China	C2	Unilateral	A
Bhi-87	B445	Dongguan, Guangdong, China	C2	Unilateral	A
Bhi-88	B435	Sanshui, Guangdong, China	C2	Unilateral	A
Bhi-89	B418	Huizhou, Guangdong, China	C2	Unilateral	A
Bhi-90	B182	Panyu, Guangdong, China	C2	Unilateral	A

Bhi-91	B497	Jiexi, Guangdong, China	C2	Unilateral	A
Bhi-92	B32	Yingde, Guangdong, China	C2	Unilateral	A
Bhi-93	B98-3	Taishan, Guangdong, China	C2	Unilateral	A
Bhi-94	B259	Guangzhou, Guangdong, China	C2	Unilateral	A
Bhi-95	B281-3	Changsha, Hunan, China	C2	Bilateral	G
Bhi-96	B518	Guangzhou, Guangdong, China	C2	Bilateral	G
Bhi-97	B509	Nanning, Guangxi, China	C2	Unilateral	A
Bhi-98	BS96	Taishan, Guangdong, China	C2	Unilateral	A
Bhi-99	B478	Guangzhou, Guangdong, China	C2	Unilateral	A
Bhi-100	B268	Foshan, Guangdong, China	C2	Unilateral	A
Bhi-101	B261	Nanchang, Jiangxi, China	C2	Unilateral	A
Bhi-102	B413	Quang Tri, Tinh, Vietnam	C2	Bilateral	A
Bhi-103	B367	Jiexi, Guangdong, China	C2	Unilateral	A
Bhi-104	B361	Guangzhou, Guangdong, China	C2	Unilateral	A
Bhi-105	B426	Sanshui, Guangdong, China	C2	Unilateral	A
Bhi-106	B94	Sanshui, Guangdong, China	C2	Unilateral	A
Bhi-107	B225	Sanshui, Guangdong, China	C2	Unilateral	A
Bhi-108	B185	Sanshui, Guangdong, China	C2	Unilateral	A
Bhi-109	B228	Sanshui, Guangdong, China	C2	Unilateral	A
Bhi-110	B48	Lianzhou, Guangdong, China	C2	Unilateral	A
Bhi-111	B45	Yingde, Guangdong, China	C2	Unilateral	A
Bhi-112	B184	Sanshui, Guangdong, China	C2	Unilateral	A
Bhi-113	B515	Guangzhou, Guangdong, China	C2	Unilateral	A
Bhi-114	B227r	Sanshui, Guangdong, China	C2	Unilateral	A
Bhi-115	B482	Jiexi, Guangdong, China	C2	Unilateral	A
Bhi-116	B522	Foshan, Guangdong, China	C2	Unilateral	A
Bhi-117	B252	Sanshui, Guangdong, China	C2	Unilateral	A
Bhi-118	B249	Sanshui, Guangdong, China	C2	Unilateral	A
Bhi-119	B481	Qingyuan, Guangdong, China	C2	Unilateral	A
Bhi-120	B480	Qingyuan, Guangdong, China	C2	Unilateral	A
Bhi-121	B510	Nanning, Guangxi, China	C2	Unilateral	A
Bhi-122	B516	Guangzhou, Guangdong, China	C2	Unilateral	A
Bhi-123	B524	Sanshui, Guangdong, China	C2	Unilateral	A
Bhi-124	B530	Sanshui, Guangdong, China	C2	Unilateral	A
Bhi-125	B451	Dongguan, Guangdong, China	C2	Unilateral	A
Bhi-126	B427	Qingyuan, Guangdong, China	C2	Unilateral	A
Bhi-127	B329	Foshan, Guangdong, China	C2	Unilateral	A
Bhi-128	B202	Taishan, Guangdong, China	C2	Unilateral	A
Bhi-129	B452	Dongguan, Guangdong, China	C2	Unilateral	A
Bhi-130	B528	Sanshui, Guangdong, China	C2	Unilateral	A
Bhi-131	B479	Guangzhou, Guangdong, China	C2	Unilateral	-
Bhi-132	B505	Changsha, Hunan, China	C2	Unilateral	A
Bhi-133	B98-1	Taishan, Guangdong, China	C2	Unilateral	A
Bhi-134	B444	Dongguan, Guangdong, China	C2	Unilateral	A
Bhi-135	B232	Lianjiang, Guangdong, China	C2	Unilateral	A
Bhi-136	B506	Changsha, Hunan, China	C2	Unilateral	A
Bhi-137	B320-3	Changsha, Hunan, China	C2	Unilateral	A
Bhi-138	B430	Guangzhou, Guangdong, China	C2	Unilateral	A

Bhi-139	B274-2	Nanning, Guangxi, China	C2	Bilateral	G
Bhi-140	B274-3	Nanning, Guangxi, China	C2	Unilateral	G
Bhi-141	B274-1	Nanning, Guangxi, China	C2	Unilateral	A
Bhi-142	B402	Nanning, Guangxi, China	C2	Bilateral	-
Bhi-143	B511	Nanning, Guangxi, China	C2	Unilateral	A
Bhi-144	B514	Nanning, Guangxi, China	C2	Unilateral	A
Bhi-145	B314	Nanning, Guangxi, China	C2	Bilateral	A
Bhi-146	B235	Shaoguan, Guangdong, China	C2	Bilateral	G

[1] W indicates wild wax gourds. L indicates landrace wax gourds. C1 and C2 indicate cultivated wax gourds.

[2] R indicates A/G base. - indicates an undetermined base.

Supplementary Table 5. Seed shape and genotype of the 42 wax gourd germplasm resources.

Number	Germplasm resource name	Seed shape	Genotype
1	B94	unilateral	unilateral
2	B96	unilateral	unilateral
3	B98-1	unilateral	unilateral
4	B102	unilateral	unilateral
5	B184	unilateral	unilateral
6	B228	unilateral	unilateral
7	B249	unilateral	unilateral
8	B277	unilateral	unilateral
9	B278	unilateral	unilateral
10	B300	unilateral	unilateral
11	B324	unilateral	unilateral
12	B327	unilateral	unilateral
13	B330	unilateral	unilateral
14	B332	unilateral	unilateral
15	B361	unilateral	unilateral
16	B367	unilateral	unilateral
17	B401	unilateral	unilateral
18	B410	unilateral	unilateral
19	B465	unilateral	unilateral
20	B480	unilateral	unilateral
21	B481	unilateral	unilateral
22	B500	unilateral	unilateral
23	B509	unilateral	unilateral
24	B214	bilateral	bilateral
25	B260-1	bilateral	bilateral
26	B235-1	bilateral	bilateral
27	B274-2	bilateral	bilateral
28	B281	bilateral	bilateral
29	B421	bilateral	bilateral
30	P74	bilateral	bilateral
31	P91	bilateral	bilateral
32	P125	bilateral	bilateral
33	S15	bilateral	bilateral
34	S16	bilateral	bilateral
35	S23	bilateral	bilateral
36	BN5-2	bilateral	bilateral
37	BN10-3	bilateral	bilateral
38	BN36-1	bilateral	bilateral
39	T7-1	bilateral	bilateral

40	T8-1	bilateral	bilateral
41	T43-1	bilateral	bilateral
42	T48-1	bilateral	bilateral
